# Antimicrobial use for selected diseases in cats in Switzerland

**DOI:** 10.1186/s12917-019-1821-0

**Published:** 2019-03-14

**Authors:** K. Schmitt, C. Lehner, S. Schuller, G. Schüpbach-Regula, M. Mevissen, R. Peter, C. R. Müntener, H. Naegeli, B. Willi

**Affiliations:** 1 Clinic for Small Animal Internal Medicine, Vetsuisse Faculty, University of Zurich, Winterthurerstrasse 260, CH-8057 Zurich, Switzerland; 2 Institute of Veterinary Pharmacology and Toxicology, University of Zurich, Winterthurerstrasse 260, CH-8057 Zurich, Switzerland; 3 Division of Small Animal Internal Medicine, Department of Clinical Veterinary Medicine, Vetsuisse Faculty, University of Bern, Bern, Switzerland; 4 Veterinary Public Health Institute (VPHI), Vetsuisse Faculty, University of Bern, Bern, Switzerland; 5 Division of Pharmacology and Toxicology, Vetsuisse Faculty, University of Bern, Bern, Switzerland

**Keywords:** Antibiotic prescription, Antimicrobial stewardship, Resistance, Guidelines, Acute upper respiratory tract disease, Feline lower urinary tract disease, FLUTD, Abscess, Infection

## Abstract

**Background:**

Antibiotic use in human and veterinary medicine is considered a main driver of antimicrobial resistance. Although guidelines to promote appropriate use of antimicrobials in veterinary patients have been developed, antibiotic overprescription is assumed to be a common problem. The goal of this study was to investigate antimicrobial use in cats in Switzerland with acute upper respiratory tract disease (aURTD), feline lower urinary tract disease (FLUTD) and abscesses, and to assess compliance of prescription with consensus guidelines. A total of 776 cases (aURTD, *n* = 227; FLUTD, *n* = 333; abscesses, *n* = 216) presented to two university hospitals and 14 private veterinary practices in Switzerland during 2016 were retrospectively evaluated. Clinical history, diagnostic work-up and antimicrobial prescription (class, dosage, duration) were assessed.

**Results:**

A total of 77% (aURTD), 60% (FLUTD) and 96% (abscesses) of the cases received antibiotic therapy; 13–24% received combination or serial therapy. The cats were treated for a median of 7 (abscesses) and 10 days (aURTD, FLUTD). Treatments with potentiated aminopenicillins (40–64%), third generation cephalosporins (25–28%), aminopenicillins (12–24%) and fluoroquinolones (3–13%) were most common. Prescriptions were judged in complete accordance with consensus guidelines in 22% (aURTD), 24% (FLUTD) and 17% (abscesses) of the cases. Antibiotics were prescribed although not indicated in 34% (aURTD), 14% (FLUTD) and 29% (abscesses) of the cases. The presence of lethargy, anorexia or fever in cats with aURTD, and the detection of bacteriuria in cats with FLUTD were significantly associated with antibiotic therapy. Although diagnostic work-up was significantly more common (aURTD: university hospitals, 58%; private practices, 1%; FLUTD: university hospitals, 92%; private practices, 27%) and the use of critically important antibiotics significantly less common at the university hospitals (aURTD, 10%; FLUTD, 14%) compared to private practices (aURTD, 38%; FLUTD, 54%), the frequency of antibiotic treatment was not different between the university hospitals and private practices.

**Conclusions:**

Our results indicate that overprescription of antibiotics in cats in Switzerland is common and accordance with guidelines is poor. The study highlights the need to promote antimicrobial stewardship in small animal medicine.

## Background

Mitigation of antimicrobial resistance has been an emerging topic that plays an important role in human and veterinary medicine. Antimicrobial resistance in bacteria is a naturally occurring phenomenon and has been subject to evolution over millions of years [1–5]. The frequent use of antimicrobials in human and veterinary medicine and in agriculture exerts an enormous selection pressure on bacterial populations and promotes the development of multidrug-resistant bacteria that can readily spread their resistance genes by various mechanisms [1–3]. Antibiotic use in veterinary medicine is discussed as one of the main drivers for resistance development. In Europe, around 8000 tons of antibiotics were sold for veterinary use in 2015, with pronounced differences between countries [6]. The amount of antibiotics used in companion animals in Europe in comparison to the amount prescribed in livestock is relatively small [6], but is not to be neglected. The close contact of pets with their owners facilitates the transmission of multidrug-resistant organisms between humans and companion animals [1, 7–14]. Furthermore, the trend for intensive medical care of dogs and cats poses a risk for nosocomial infections [15–18] and is associated with an increasing number of geriatric and immunosuppressed patients that are highly susceptible to infections with multidrug-resistant bacteria.

Based on recent data, Centres for Disease Control and Prevention estimates that approximately 30 –50% of antibiotic prescriptions in humans are unnecessary [19, 20]. Surveys describing antimicrobial use in dogs and cats revealed that antibiotics are frequently prescribed, in particular beta-lactam antibiotics [21–30], and that cats are especially exposed to the critically important third generation cephalosporins [21–25, 30–33]. Most previous studies performed in dogs and cats were based on questionnaires presenting hypothetical scenarios that have been sent out to veterinarians [22, 23, 28, 33–37]. These studies are commonly hampered by a selection bias, recall bias and prevarication bias, and the answers given do not necessarily reflect the actual prescribing practice. Some studies analyzed pharmacy records [38, 39] and veterinary or pet insurance databases [21, 22, 24–27, 29, 30, 32] but only few studies evaluated whether prescriptions practice was in accordance with relevant guidelines [24, 26, 27, 33, 34].

The aims of this study were to investigate the antimicrobial prescribing practice in Switzerland for indications in cats with frequent use of antibiotics, i.e., in cases of acute upper respiratory tract disease (aURTD), feline lower urinary tract disease (FLUTD) as well as abscesses, and to evaluate to what extend the prescriptions comply with recently established consensus guidelines [40, 41]. The a priori compliance with the proposed guidelines was evaluated in this study to lay the basis to monitor, in a next step, the impact of these guidelines on antimicrobial prescription patterns in Switzerland.

## Results

### Case characteristics

A total of 776 cats were included in the study. Case characteristics are shown in Table 1. A detailed overview of clinical symptoms, diagnostic procedures and antimicrobial prescription for each indication is given in Tables 2, 3 and 4. Cats with aURTD were significantly younger (median age: 3 years) than cats with FLUTD (median age: 8 years, *p* < 0.001) or cats with abscesses (median age: 7 years; *p* < 0.001) and more likely to be intact (aURTD and FLUTD: *p* < 0.001, aURTD and abscesses: *p* < 0.001). Furthermore, the cats presented to the university hospitals were more often pretreated with antibiotics (aURTD, 30%; FLUTD, 18%) and hospitalized (aURTD, 72%; FLUTD, 69%) when compared with the cases in private practices (pretreatment: aURTD, 4%; FLUTD, 2%; hospitalization: aURTD, 3%; FLUTD, 15%). The frequency of antibiotic prescription differed between the indications (percentage of cases treated: aURTD, 77%; FLUTD, 60%; abscesses, 96%; *p* < 0.001), but was not significantly associated with breed, age or sex of the cats.

**Table 1:** Characteristics of cats with aURTD^a^, FLUTD^b^, and abscesses presented to university hospitals or private practices

Parameter		aURTDª			FLUTD^b^			Abscesses
		University hospitals	Private practices	*p*-value	University hospitals	Private practices	*p*-value	Private practices
Total number of cases		*n* = 43	*n* = 184		*n* = 130	*n* = 203		*n* = 216
Sex	Female	17 (40%)	82 (45%)	n.s.^c^	43 (33%)	97 (48%)	0.006	64 (30%)
	Male	24 (55%)	97 (53%)		87 (67%)	104 (51%)		148 (68%)
	Unknown	2 (5%)	5 (1%)	n.a.^d^	0 (0%)	2 (1%)	n.a.^d^	4 (2%)
Age (years)	Median (range; CI^e^)	5 (0.042 –16; 2, 9)	2 (0.06–19; 1, 4)	n.s.^c^	7, (1–21; 7, 9)	8 (0.17–20; 6, 9)	n.s.^c^	7 (1–18; 6, 8)
Breed	Purebred	11 (26%)	33 (18%)	n.s.^c^	36 (28%)	33 (16%)	0.026	14 (6%)
	Mixed breed	29 (67%)	143 (78%)		90 (69%)	157 (77%)		190 (88%)
	Unknown	3 (7%)	8 (4%)	n.a.^d^	4 (3%)	13 (7%)	n.a.^d^	12 (6%)
Vaccinated	Yes	14 (33%)	28 (15%)	n.s.^c^	n.a.^d^	n.a.^d^	n.a.^d^	n.a.^d^
	No	17 (39%)	50 (27%)		n.a.^d^	n.a.^d^		n.a.^d^
	Unknown	12 (28%)	106 (58%)	n.a.^d^	n.a.^d^	n.a.^d^	n.a.^d^	n.a.^d^

ªaURTD, acute upper respiratory tract disease; ^b^FLUTD, feline lower urinary tract disease; ^c^n.s., not significant; ^d^n.a., not applicable; ^e^CI, confidence interval

**Table 2:** Diagnostic work-up and antibiotic prescription in aURTD^a^ cases presented to university hospitals or private practices

Parameter		University hospitals	Private practices	*p*-value
Total number of cases		*n* = 43	*n* = 184	
Diagnostic work-up with PCR^b^	Yes^c^	25 (58%)	2 (1%)	<0.001
At least one of the symptoms listed in the guidelines^d^	Yes^c^	29 (68%)	37 (21%)	<0.001
	Unknown	1 (3%)	42 (23%)	
Hospitalization	Yes^c^	31 (72%)	5 (3%)	<0.001
Pre-treated with antibiotics	Yes^c^	13 (30%)	8 (4%)	<0.001
	Unknown	2 (5%)	4 (2%)	
Antibiotic therapy	Yes^c^	31 (72%)	144 (78%)	n.s.^e^
Antibiotic classes	Potentiated aminopenicillins	28 (90%)	42 (29%)	<0.001
	Third generation cephalosporins	2 (6%)	47 (33%)	0.002
	Aminopenicillins	1 (3%)	40 (28%)	0.002
	Tetracyclines	2 (6%)	26 (18%)	n.s.^e^
	Fluoroquinolones	1 (3%)	6 (4%)	n.s.^e^
	Amphenicols	0 (0%)	3 (2%)	n.s.^e^
	Macrolides	0 (0%)	3 (2%)	n.s.^e^
	First generation cephalosporins	0 (0%)	1 (1%)	n.s.^e^
	Penicillins	0 (0%)	1 (1%)	n.s.^e^
Combination or serial therapy^f^	Yes^c^	4 (13%)	21 (15%)	n.s.^e^
Critically important antibiotic^f^	Yes^c^	3 (10%)	55 (38%)	0.001
Duration of therapy (days)	Median (range)	12 (4–27)	10 (4–37)	n.s.^e^
Justification score^f^	1	12 (28%)	37 (20%)	n.s.^e^
	2	1 (2%)	3 (2%)	n.s.^e^
	3	26 (61%)	22 (12%)	<0.001
	4	3 (7%)	80 (43%)	<0.001
	Judgement not possible	1 (2%)	42 (23%)	0.001

^a^aURTD, acute upper respiratory tract disease; ^b^PCR, polymerase chain reaction; ^c^Values for the category “no” (reference group) are not shown; ^d^Poor general condition, fever, lethargy and/ or anorexia; ^e^n.s., not significant; ^f^As defined in methods

**Table 3:** Diagnostic work-up and antibiotic prescription in FLUTD^a^ cases presented to university hospitals or private practices

Parameter		University hospitals	Private practices	*p*-value
Total number of cases		*n* = 130	*n* = 203	
Urine analysis performed	Yes^b^	119 (92%)	55 (27%)	<0.001
Sediment analysis	Yes^b^	93 (72%)	50 (25%)	<0.001
Bacterial culture	Yes^b^	113 (87%)	20 (10%)	<0.001
Confirmed bacterial etiology^c^	Yes^b^	45 (35%)	16 (8%)	<0.001
Hospitalization	Yes^b^	90 (69%)	30 (15%)	<0.001
Pre-treated with antibiotics	Yes^b^	23 (18%)	4 (2%)	<0.001
	Unknown	5 (4%)	3 (2%)	
Antibiotic therapy	Yes^b^	85 (65%)	115 (57%)	n.s.^d^
Antibiotic classes	Potentiated aminopenicillins	71 (84%)	50 (57%)	<0.001
	Third generation cephalosporins	7 (8%)	44 (38%)	<0.001
	Fluoroquinolones	5 (6%)	20 (17%)	0.017
	Aminopenicillins	1 (1%)	22 (19%)	<0.001
	First generation cephalosporins	5 (6%)	1 (1%)	n.s.^d^
	Amphenicols	1 (1%)	0 (0%)	n.s.^d^
	Tetracyclines	1 (1%)	0 (0%)	n.s.^d^
Combination or serial therapy^e^	Yes^b^	6 (7%)	20 (17%)	n.s.^d^
Critically important antibiotic^e^	Yes^b^	12 (14%)	62 (54%)	<0.001
Duration of therapy (days)	Median (range)	13 (1 –56)	9 (1 –42)	0.012
Justification score^e^	1	57 (44%)	24 (12%)	<0.001
	2	1 (1%)	0 (0%)	n.s.^d^
	3	22 (17%)	9 (4%)	<0.001
	4	39 (30%)	11 (6%)	<0.001
	Judgement not possible	11 (8%)	159 (78%)	<0.001

^a^FLUTD, feline lower urinary tract disease; ^b^Values for the category “no” (reference group) are not shown; ^c^Defined as either positive urine sediment analysis or positive bacterial culture; ^d^n.s., not significant; ^e^As defined in methods

**Table 4:** Clinical signs, wound treatment and antibiotic prescription in cases with abscesses presented to private practices

Parameter		Private practices
Total number of cases		*n* = 216
At least one of the symptoms listed in the guidelines^a^	Yes^b^	65 (30%)
	Unknown	85 (39%)
Local wound treatment	Yes^b^	156 (72%)
	Unknown	12 (6%)
Drain placement	Yes^b^	33 (15%)
Antibiotic therapy	Yes^b^	207 (96%)
Antibiotic classes	Potentiated aminopenicillins	132 (64%)
	Third generation cephalosporins	52 (25%)
	Aminopenicillins	50 (24%)
	First generation cephalosporins	12 (6%)
	Fluoroquinolones	5 (3%)
	Lincosamides	4 (2%)
	Penicillins	1 (1%)
Combination or serial therapy^c^	Yes^b^	48 (24%)
Critically important antibiotic^c^	Yes^b^	57 (28%)
Duration of therapy (days)		7 (1 –24)
Justification score^c^	1	36 (17%)
	2	16 (7%)
	3	14 (7%)
	4	65 (30%)
	Judgement not possible	85 (39%)

^a^Signs of generalization, poor general condition, severely contaminated wounds, and/ or proximity to delicate tissues; ^b^Values for the category “no” (reference group) are not shown; ^c^As defined in methods

### Antibiotic prescription for aURTD

Of 227 cats with aURTD, 175 (77%) received antibiotic therapy with the following substance classes: potentiated aminopenicillins (40%), third generation cephalosporins (28%), aminopenicillins (24%), tetracyclines (16%), fluoroquinolones (4%), amphenicols (2%), macrolides (2%), first generation cephalosporins and penicillins (1% each); 15% of the cases received combination or serial therapy. The antimicrobial combinations used were potentiated or non-potentiated aminopenicillins together with fluoroquinolones, first generation cephalosporins, tetracyclines, amphenicols or third generation cephalosporins. One cat received a triple therapy consisting of an aminopenicillin, a fluoroquinolone and a tetracycline. The cats were treated for 4 to 37 days (median of 10 days). Antibiotic therapy was significantly associated with the indications listed in the guidelines (presence of lethargy, anorexia or fever, *p* = 0.002). The treatment decision was judged as being compliant with the guidelines (justification score-1, JS-1) in 49 cases (22%) and not in accordance with the guidelines in 135 cases (59%; JS-2: *n* = 4, 2%; JS-3: *n* = 48, 21%; JS-4, *n* = 83, 36%). In the 83 cases where a complete discrepancy with the guidelines was found (JS-4), antibiotic prescription although not indicated (overprescription) occurred in 78 cases (94%) whereas 5 cases (6%) did not receive antibiotics despite indicated in the guidelines. The lack of information on the presence or absence of disease symptoms as listed in the guidelines precluded judgment in 43 cases (19%). Judgement of antimicrobial prescription was significantly more often not possible in private practices compared to the university hospitals (*p* = 0.001).

The diagnostic work-up and antimicrobial prescription patterns differed between private practices and university hospitals (Table 2). The aURTD cases were significantly more frequently tested by PCR for the presence of respiratory pathogens at the university hospitals (58%) compared to private practices (1%). The choice of antibiotic was significantly more often in disagreement with the guidelines (JS-3) at the university hospitals (61%) than at the private practices (12%). This was mainly due to the more common use of potentiated aminopenicillins (university hospitals, 90%; private practices, 29%) and the less common use of aminopenicillins (university hospitals, 3%; private practices, 28%) at the university hospitals compared to private practices. On the other hand, the use of critically important antibiotics was significantly more common in private practices (38%; university hospitals, 10%). The decision to use antibiotics for treatment was significantly more often in disagreement with the guidelines (JS-4) in the private practices (43%) when compared to the university hospitals (7%).

### Antibiotic prescription for FLUTD

Of 333 cats with FLUTD, 200 cases (60%; 56 with bacterial cystitis, 144 with other/unknown diagnosis) received antibiotic therapy with the following substance classes: potentiated aminopenicillins (61%), third generation cephalosporins (26%), fluoroquinolones (13%), aminopenicillins (12%), first generation cephalosporins (3%), amphenicols (1%) and tetracyclines (1%); 13% received combination or serial therapy. For combination therapy, potentiated or non-potentiated aminopenicillins together with fluoroquinolones or third generation cephalosporins were used. The cats were treated for 1 to 56 days (median of 10 days). The presence of bacteriuria was significantly associated with antibiotic therapy (*p* < 0.001). The treatment decision was judged as being compliant with the guidelines (JS-1) in 81 (24%) and not in accordance with the guidelines in 82 (25%) cases (JS-2: *n* = 1, 1%; JS-3: *n* = 31, 9%; JS-4: *n* = 50, 15%). In the 50 cases with complete discrepancy to the guidelines (JS-4), antibiotics were prescribed although not indicated (overprescription) in 47 cases (94%) and cats were not treated with antibiotics despite indicated in the guidelines in 3 cases (6%). Inadequate diagnostic work-up (154 out of 170 cases, 91%) was the main reason to preclude judgment in 170 cases (51%).

Diagnostic work-up and antimicrobial prescription patterns were again different between the university hospitals and private practices (Table 3). Urine sediment analysis or bacterial culture were significantly more commonly performed at the university hospitals (92%) compared to private practices (27%). When antimicrobial prescription at the university hospitals was compared to the private practices, prescription was significantly more often graded as JS-1 (complete agreement with the guidelines; university hospitals, 44%; private practices 12%), JS-3 (choice of antimicrobial different from the guidelines; university hospitals, 17%; private practices 4%) and JS-4 (complete discrepancy with the guidelines; university hospitals, 30%; private practices 6%). The use of critically important antibiotics was significantly more common in private practices (54%) compared to the university hospitals (14%). Furthermore, judgment of antimicrobial prescription was significantly more often not possible in private practices (78%; university hospitals, 8%).

### Antibiotic prescription for abscesses

Of 216 cats with abscesses, 207 cats (96%) received antibiotic therapy with the following substance classes: potentiated aminopenicillins (64%), third generation cephalosporins (25%), aminopenicillins (24%), first generation cephalosporins (6%), fluoroquinolones (3%), lincosamides (2%) and penicillins (1%); 24% received combination or serial therapy. Combination therapy was uncommon (3 cases) and antimicrobial combinations used were potentiated or non-potentiated aminopenicillins together with fluoroquinolones or third generation cephalosporins. The cats were treated for 1 to 24 (median 7) days. Local wound treatment was carried out in 156 of 216 cases (72%) and drains were placed in 33 of 216 cases (15%). Antibiotic therapy was not associated with any of the symptoms listed in the guidelines, i.e., signs of generalization, poor general condition, severely contaminated wounds, and/ or proximity to delicate tissues. Antimicrobial therapy was judged in accordance with the guidelines (JS-1) in 36 (17%) and not in accordance with the guidelines in 95 (44%) cases (JS-2: *n* = 16, 7%; JS-3: *n* = 14, 7%; JS-4: *n* = 65, 30%). In the 65 cases with complete discrepancy to the guidelines (JS-4), antibiotics were prescribed without indication (overprescription) in 63 cases (97%) and cats were not treated with antibiotics despite indicated in the guidelines in 2 cases (3%). Assessment of prudent use was not possible for 85 cases (39%).

## Discussion

The results of this study indicate that overprescription of antibiotics in cats in Switzerland with aURTD, FLUTD and abscesses is very common. When prescription was compared to the consensus guidelines, 14–34% of all cases received antibiotics although not indicated; when only the cases were considered for which judgment of prudent use was possible, the rate of antibiotic overprescription was even higher (29–48%). The diagnostic work-up was more elaborate at the university hospitals, and critically important antibiotics were less commonly prescribed at the universities, but the prudent use pattern of prescriptions was not clearly superior when compared to private practices. This was mainly due to the very common use of potentiated aminopenicillins instead of non-potentiated aminopenicillins at the universities. On the other hand, the quality of antimicrobial prescription could often not be judged in the cases from private practices because the diagnostic work-up or the symptoms of the patients were not documented. The common discrepancy of antimicrobial prescription with recently established consensus guidelines [40, 41] at the two university hospitals is surprising considering that senior clinicians of these hospitals were involved in the drafting of the guidelines. The overall frequency of antimicrobial treatment was also not different at the university hospitals compared to private practices. However, our data indicates that the animals presented to the university hospitals were more often pretreated or hospitalized, and could thus have been in a more debilitated condition.

Only 17 –24% of the treatment decisions in this study were classified as JS-1 and therefore in complete accordance with the consensus guidelines. Recent studies in dogs and cats have reported an overall agreement of 0–69% with published guidelines [24, 27, 33, 34]. This overall low accordance raises the question of whether the proposed guidelines cannot be implemented in clinical practice, for example, due to poor market availability of appropriate antibiotic formulations, or whether the content is not well disseminated among veterinary practitioners.

The critically important antibiotics used in cats in this study were mostly third generation cephalosporins as well as fluoroquinolones. Third generation cephalosporins were the second most frequently prescribed antibiotic class and were used in 25–28% of the cases. This mirrors the results obtained in previous studies [21–25, 31–33] and could likely be explained by the convenient application (as a single subcutaneous injection) and the long dosing interval (2 weeks) of the authorized product in Switzerland (cefovecin, Convenia®, Zoetis, Delémont, Switzerland). A previous study evaluating electronic health records found that inability to orally medicate the cat was the most common reason given for prescribing cefovecin [42]. An online survey in veterinarians in Switzerland also revealed that the route of application was the most important factor in the choice of antimicrobial therapy in cats [43]. In our study, the prescription of critically important antibiotics was significantly more frequent in private practices compared to the university hospitals. This observation supports the hypothesis that the workplace environment is an important factor determining treatment decisions and antimicrobial use [44]. University hospitals, as training centers, may have stronger restrictions for the use of critically important antibiotics: one of the two university hospitals of this study completely forbids the use of third generation cephalosporins in its patients. On the other hand, the cats at the university hospitals were more commonly hospitalized compared to the cases in private practices, thus allowing for parenteral medication and avoiding the problem of oral application of the antibiotic drug.

Antimicrobial prescription in the absence of proper diagnostic work-up was very common in this study. In only 40% of the FLUTD cases overall, and in only 10% of the FLUTD cases in private practices, bacterial culture and susceptibility testing were carried out. In a previous study based on a questionnaire, 32.5% of companion animal practitioners in Europe reported that they frequently undertake antimicrobial susceptibility testing whereas 9.1% never demand such tests [45]. In another survey from Italy, 91% of the practitioners reported to carry out microbiological analysis, although only 20% reported to do so frequently [35]. Our results indicate that these data based on questionnaires are probably too optimistic and that bacterial culture, an essential diagnostic work-up step for cats with FLUTD, is rarely performed in private practices. In contrast, bacterial culture was performed in 87% of the FLUTD cases presented to the university hospitals, although this did not result in a less frequent prescription of antimicrobials. Interestingly, 56% of the FLUTD cases at the universities received antibiotic therapy despite the absence of bacteria in urine culture. A total of 20% of these cats were pretreated with antibiotics which could have affected the interpretation of a negative bacterial culture result. Also many of these cats suffered from urinary tract obstruction and antibiotic therapy was initiated after removal of the indwelling urinary catheter.

The trend towards more diagnostic testing at the university hospitals is also demonstrated by a more frequent use of PCR for the detection of feline calicivirus (FCV) and feline herpesvirus-1 (FHV) in cases with aURTD. These tests can be useful to support a diagnosis of viral infection and to initiate supportive measures such as the prescription of famciclovir in the case of FHV infection, and thus reduce the use of antibiotics [46]. In this study, the detection of FCV and FHV did not affect the frequency of antibiotic prescription. Overprescription of antibiotics in cats with aURTD was common: although only 29% of the cats showed symptoms that would have justified an antibiotic therapy according to the consensus guidelines, 77% of the cases received antibiotic treatment. Potentiated aminopenicillins were most often prescribed at the university hospitals, while third generation cephalosporins and aminopenicillins were most commonly used in private practices. A study revealed that amoxicillin with clavulanic acid is not superior to doxycycline when treating cats with signs of acute respiratory tract disease [47]. Our data, however, indicate that the cases presented to the university hospitals were in a more debilitated condition, because 72% of the cats with aURTD were hospitalized in comparison to 3% in private practices. Furthermore, 68% of the cats presented to university hospitals had symptoms listed in the guidelines, while at the private practices only 21% of the cats were reported to show a poor general condition, fever, lethargy and/or anorexia. The more compromised clinical condition of the patients at university hospitals could explain the common use of potentiated aminopenicillins instead of doxycycline due to the lack of a licensed injectable doxycycline preparation in Switzerland. However, potentiated instead of non-potentiated aminopenicillins were almost exclusively used at the universities. Non-potentiated and potentiated aminopenicillins are often used interchangeably although it has been shown that the use of clavulanic acid may increase AmpC-mediated resistance causing inducible organisms to become insensitive to 1st to 3rd generation cephalosporins [48, 49]. The frequent use of potentiated aminopenicillins instead of non-potentiated aminopenicillins in this study could also be due to the better availability of these products on the market, since they make up the largest part of antimicrobial compounds licensed for cats in Switzerland [50].

A total of 96% of the cats with abscesses received antibiotic treatment but only 30% of the cats presented symptoms that, according to the guidelines, would justify an antibiotic therapy. Our findings are in line with results from previous studies where frequency of antibiotic prescriptions for skin diseases such as wounds or abscesses ranged from 90 to 97% [24, 32]. In children, antibiotics might sometimes be applied instead of local wound drainage to avoid anesthesia or sedation [51]. However, 72% of the cats in this study received local wound treatment and passive drains were placed in 15% of the cats. It can be assumed that, in many of these cases, antibiotics were supplemented as a preventative measure. Studies from human medicine showed that appropriate drainage of the abscess is important and that antibiotic treatment may not be necessary [52–55]. Several guidelines for small animals state that an antibiotic treatment is not indicated if the abscess is well-defined and the animal is in a good general condition [56–58].

The present study has some limitations. The insufficient documentation in the databases limited the information available for review. The presence of bacteria in urine sediment analysis of an aseptically collected urine sample was considered appropriate to confirm a bacterial etiology in cases with FLUTD, although this is considered insufficient diagnostic work-up according to some guidelines due to the variable quality of interpretation, the risk of stain contamination as well as the possibility of false positive results [57, 59]. In a recent study, overall accuracy of in-house microscopic evaluation for bacteriuria in primary practice was only 64.5% when comparing results to reference bacterial cultures [60]. We decided to consider these results since former studies have reported accuracies of urine sediment analysis of 97–98% when performed by experienced laboratory personal [61–64]. Furthermore, the generally low prevalence of bacterial cystitis in cats should not result in many false positive results [65–67].

The assessment score used in this study leaves a margin of interpretation and the justification of antimicrobial prescription was based on consensus guidelines released in Switzerland. Results could differ to some extent when comparing prescription to guidelines from other countries. Furthermore, the limited number of cases included per practice did also not allow for a statistical analysis at a single practice level. Also, there could be a selection bias since the participation in this study was on a voluntary basis and the enrolled practices might have been more aware of antimicrobial resistance and more likely to prescribe antibiotics prudently.

## Conclusions

The present study highlights the need to promote antimicrobial stewardship in small animal medicine and to implement effective intervention strategies. Particular attention should be paid to the education of veterinarians, the propagation of diagnostic work-up and the need for proper documentation to justify antibiotic treatment. Antimicrobial stewardship at universities should be urgently advanced as they serve as role models for veterinary practice. Developments on the market to provide small spectrum antibiotics for convenient application would be of particular importance in cats since the route of application is a major factor in the choice of antimicrobials in this species. Such new products will contribute to ensure that effective antimicrobials remain available in the future to combat bacterial infections in human and veterinary medicine.

## Methods

Cases presented between January 1st and December 31st 2016 to the two Swiss university teaching hospitals for small animals (Vetsuisse Faculty Bern and Zurich) as well as to fourteen private veterinary practices across Switzerland were included. The private practices participated on a voluntary basis following a nationwide call. In order to identify patients matching the inclusion criteria (Table 5), the electronic records were scanned for predetermined search terms (Table 5) using search functions provided by the particular software. For practical reasons, only private practices using either OblonData® (Amacker&Partner Informatik AG, Zurich, Switzerland) or Diana SUISSE® (Diana Software AG, Zurich, Switzerland) were enrolled. A full text search was conducted and the matches were manually reviewed. All cases from the two university hospitals matching the criteria were included. From each private practice, 16 cases per indication that matched the criteria were randomly selected via the sampling function of Microsoft® Excel. In the eight private practices where less than 16 cases per indication were found, all cases were included. The number of cases was balanced by limiting the number to 16 per practice to avoid overrepresentation of larger private practices. Although not all cases were included, the random selection should ensure that the results remain representative.

**Table 5:** Inclusion and exclusion criteria and search terms for aURTD^a^, FLUTD^b^ and abscesses

Indication	Inclusion criteria	Exclusion criteria	Search terms
aURTDª	Nasal discharge with infectious or unknown etiology lasting no longer than 2 weeks	Evidence of fungal infection, neoplasia or involvement of the lower respiratory tract	Upper respiratory tract infection, rhinotracheitis, rhinitis, sinusitis, nasal discharge, sneezing, coughing, stridor, dyspnea, tachypnea, cat flu, herpes, calici, mycoplasma, chlamydia, laryngitis
FLUTD^b^	Stranguria, pollakiuria, periuria, pigmenturia or dysuria and a diagnosis of bacterial cystitis, bladderstones, urethrastones, urethral plugs, idiopathic cystitis or cystitis of unknown origin	Involvement of the upper urinary tract	Lower urinary tract disease, FLUTD^b^, pollakiuria, polyuria, anuria, stranguria, dysuria, hematuria, bloody urine, urinary stones, bladder stones, urolithiasis, concrements, cystitis, urethra obstruction, urinary tract infection, UTI, urinary incontinence
Abscess	Bite abscesses or abscesses of unknown origin	Anal gland abscesses, tooth root abscesses, foreign body abscesses	Abscess, bite wound, bite, pus

ªaURTD, acute upper respiratory tract disease; ^b^FLUTD, feline lower urinary tract disease

Signalment, vaccination status, clinical history, reports on clinical examination, antibiotic pretreatment, diagnostic work-up, comorbidities, hospitalization and antimicrobial therapy (substance class, dose, frequency of application and duration of therapy) were retrieved from the medical records. The evaluated diagnostic work-up for aURTD included PCR for FCV and FHV; for FLUTD, urine sediment analysis and urine bacterial culture were assessed. Bacteriuria was defined as the presence of bacteria in the urine sediment analysis or in the bacterial culture from an aseptically collected urine sample (cystocentesis or catheterization). Complicated urinary tract infections were defined as infections that were caused by anatomical or functional changes or a comorbidity, that predispose the patient to persistent or recurrent infections or treatment failure [68]. Critically important antibiotics comprised third or higher generation cephalosporins, quinolones, macrolides, ketolides, glycopeptides and polymyxins [69]. Combination therapy was defined as the prescription of two or more antibiotic classes at the same time; serial therapy as the prescription of one antibiotic class followed by a different antibiotic class. Antimicrobial prescription was compared with the consensus guidelines summarized in Table 6 using a previously published JS with modifications shown in Table 7 [70]. The guidelines were published in December 2016 [40] and can be accessed online as the AntibioticScout tool [71]. The present study evaluated antimicrobial prescription prior to the implementation of the guidelines to use this data as a baseline for follow-up studies on the influence of the guidelines on antimicrobial prescription in Switzerland.

**Table 6:** Consensus guidelines [40, 41] used to evaluate prudent use of antimicrobials

Indication	Comment	Antibiotic	Dosage (mg/kg)	Application frequency	Treatment duration (days)
aURTD^a^	Antibiotic therapy is only indicated if poor general condition, fever, lethargy and/or anorexia are present	Doxycycline	10 / 5	SID^b^/BID^c^	5–14
		Amoxicillin	15–20	BID^c^/TID^d^	5–14
FLUTD^e^	Complicated UTI^f^ are defined as infections that are caused by anatomical or functional changes or disorders of the immune system	Uncomplicated UTI^f^:			
		Amoxicillin	11–15	BID^c^/TID^d^	5–7
		Complicated UTI^f^:			
		Amoxicillin/Clavulanic acid	12.5–20	BID^c^/TID^d^	5–28
Abscess	Antibiotic therapy is only indicated if signs of generalization, poor general condition, severely contaminated wounds, and/or proximity to delicate tissues (e.g., joints) are present	Amoxicillin	15–20	BID^c^	5–7
		Amoxicillin/Clavulanic acid	12.5–20	BID^c^	5–7
		Cefalexin	20–30	BID^c^/TID^d^	5–7
		Clindamycin	10–15	BID^c^	5–7
		Cefazolin	20	BID^c^	5–7

^a^aURTD, acute upper respiratory tract disease; ^b^SID, once daily; ^c^BID, twice daily; ^d^TID, three times daily; ^e^FLUTD, feline lower urinary tract disease; ^f^UTI, urinary tract infection

**Table 7:** Justification score (JS^a^) used to compare antimicrobial prescription to consensus guidelines

Justification score	Explanation
1	Indication, antimicrobial class, dose and treatment duration in accordance with the guidelines
2	Antibiotic therapy indicated but duration^b^ and/or dose^c^ of treatment not in accordance with the guidelines
3	Antibiotic therapy indicated but antimicrobial class not in accordance with the guidelines^d^
4	Complete discrepancy with the guidelines, i.e. antibiotics were prescribed without indication or, conversely, antibiotics were not prescribed despite being indicated

^a^Modified from De Pestel et al., 2014 [70]; ^b^A margin of 1 day shorter or longer was tolerated; ^c^A deviation of up to 20% above or below the recommended dose was accepted; ^d^Each case was scored only once. If the dose or duration of treatment as well as the antibiotic class were deviating from the guidelines, the case was categorized as JS-3

For statistical analysis, the commercially available SPSS® software (SPSS Inc., IL, USA) was used. Descriptive statistics and comparisons of groups were conducted. Because the continuous variables were not normally distributed, the Mann Whitney U Test was used to compare the median age as well as the duration of therapy between the university hospitals and private practices. For the median age, 95% confidence intervals (CI) were calculated. The Chi square test was performed for comparison of categorical variables (case characteristics, diagnostic work-up, hospitalization, antibiotic pretreatment and antibiotic prescription) between university hospitals and private practices; frequency of antibiotic therapy between the indications; association of symptoms listed in the guidelines (for aURTD and abscesses) or the presence of bacteriuria (for FLUTD) with antibiotic therapy. The level of significance was set at *p* < 0.05. For the comparison of the justification scores between university hospitals and private practices the Chi square test was performed and the significance level was adapted for multiple tests using the Bonferroni correction.

## Abbreviations

aURTD: acute upper respiratory tract disease
BID: twice daily
CI: confidence interval
FCV: Feline Calicivirus
FHV: Feline Herpesvirus-1
FLUTD: feline lower urinary tract disease
JS: justification score
n.a.: not applicable
n.s.: not significant
PCR: polymerase chain reaction
SID: once daily
TID: three times daily
vs: versus
